# In vivo Brain Estrogen Receptor Expression By Neuroendocrine Aging And Relationships With Gray Matter Volume, Bio-Energetics, and Clinical Symptomatology

**DOI:** 10.21203/rs.3.rs-2573335/v1

**Published:** 2023-02-27

**Authors:** Lisa Mosconi, Steven Jett, Matilde Nerattini, Caroline Andy, Camila Boneu Yepez, Camila Zarate, Caroline Carlton, Vibha Kodancha, Eva Schelbaum, Schantel Williams, Silky Pahlajani, Susan Loeb-Zeitlin, Yelena Havryliuk, Randolph Andrews, Alberto Pupi, Douglas Ballon, James Kelly, Joseph Osborne, Sadek Nehmeh, Matthew Fink, Valentina Berti, Dawn Matthews, Jonathan Dyke, Roberta Diaz Brinton

**Affiliations:** Weill Cornell Medicine; Weill Cornell Medicine; Weill Cornell Medicine; Weill Cornell Medicine; Weill Cornell Medicine; Weill Cornell Medicine; Weill Cornell Medicine; Weill Cornell Medicine; Weill Cornell Medicine; Weill Cornell Medicine; Weill Cornell Medicine; Weill Cornell Medicine; Weill Cornell Medicine; ADM Diagnostics; University of Florence; Weill Medical College of Cornell University; Weill Cornell Medicine; Weill Cornell Medicine; Weill Cornell Medicine; Weill Cornell Medicine; University of Florence; ADM Diagnostics; Weill Cornell Medicine; University of Arizona

## Abstract

17β-estradiol,the most biologically active estrogen, exerts wide-ranging effects in brain through its action on estrogen receptors (ERs), influencing higher-order cognitive function and neurobiological aging. However, our knowledge of ER expression and regulation by neuroendocrine aging in the living human brain is limited. This *in vivo* multi-modality neuroimaging study of healthy midlife women reveals progressively higher ER density over the menopause transition in estrogen-regulated networks. Effects were independent of age and plasma estradiol levels, and were highly consistent, correctly classifying all women as being post-menopausal or not. Higher ER density was generally associated with lower gray matter volume and blood flow, and with higher mitochondria ATP production, possibly reflecting compensatory mechanisms. Additionally, ER density predicted changes in thermoregulation, mood, cognition, and libido. Our data provide evidence that ER density impacts brainstructure, perfusion and energy production during female endocrine aging, with clinical implications for women’s health.

## Introduction

Estrogen is a class of steroid hormones that play a key role in the development and regulation of female reproductive system. Additionally, burgeoning evidence documents that 17β-estradiol (E2), the most biologically active estrogen, exerts wide-ranging effects on neurological and cognitive functions^1,2^, as well as neurodevelopmental and neurodegenerative processes^2,3^.

E2 signals through estrogen receptors (ERs) that are ubiquitously distributed in brain^1,2^ where they coordinate neuroprotective signaling cascades^4^ and modulate cerebral blood flow, energy metabolism, inflammation, and oxidative processes^5,6^. This is fundamental for humans, as all women experience a dramatic drop in circulating estradiol as they undergo menopause either through the natural endocrine aging process or through medical intervention^7^. Given the extraordinary integrative power of E2 for brain function^1,2^, it is not surprising that many of the symptoms of menopause are neurological in origin, manifesting as changes in thermoregulation, mood, sleep and cognition^3,8^.

Hypoestrogenic post-menopausal women are also more vulnerable to brain injury, affective disorders, and neurodegenerative conditions such as Alzheimer's disease (AD)^9^, which are influenced by ER regulation^4^. In translational brain imaging studies, the menopause transition is associated with reduced gray matter volume (GMV) and glucose metabolism^10-12^, as well as emergence of amyloid-beta (Aβ) plaques, a hallmark of AD, in midlife women^11,13^. Post-menopausal GMV and metabolic declines are attenuated by estrogen therapy^14,15^, suggesting that ER-mediated neurological processes retain dynamic properties well into menopause.

Despite preclinical evidence of the importance of ERs for neural function and the increasing use of estrogen therapies in clinical practice, our direct knowledge of ER activity in the human brain is very limited.

Positron emission tomography (PET) with selective ER ligands is the only technique currently available that enables *in vivo* assessment of ER expression. 16α-^18^F-fluoro-17β-estradiol (^18^F-FES) is the most utilized ER ligand in oncology, exhibiting high binding affinity for ERs, especially ERα^16^, and excellent signal correlation with ER expression in tumors^17,18^. ^18^F-FES studies of brain ER expression are instead, scarce. Animal studies indicate high ^18^F-FES uptake in brain ER-rich regions, mainly pituitary gland and hypothalamus^19-21^ and lower yet measurable signal in preoptic area, striatum, amygdala, hippocampus, and cortex^22^. This pattern is consistent with the reported distribution of ERs *in vitro* and *ex vivo*^1,2^. Currently, the only ^18^F-FES PET study of human brain was conducted in breast cancer patients^21^. This study provided important evidence of high tracer uptake in pituitary, with trends toward higher levels in post-menopausal as compared to pre-menopausal patients^21^. A few case studies also reported specific ^18^F-FES signal in pituitary and hypothalamus of breast cancer patients and post-menopausal controls^23,24^.

Herein, we took a translational approach to systematically investigate brain ER density and its modulation by neuroendocrine aging using *in vivo* ^18^F-FES PET in healthy midlife women. Additionally, we: (i) conducted multi-modality brain imaging to test for associations of ER density with gray matter volume (GMV), cerebral blood flow (CBF) and mitochondrial adenosine triphosphate (ATP) production in estrogenic regions; and (ii) examined the relationships between ER density and menopausal symptoms of neurological origin.

## Results

### ER density by menopause status

To characterize ER distribution and modulation by neuroendocrine aging, we performed ^18^F-FES PET imaging on midlife women, ages 40-65 years, balanced by menopausal stage: pre-menopause (scanned at midcycle), peri-menopause, and post-menopause. Demographic information on the sample is provided in the Online Methods and **Supplementary Table 1**. Image analysis was performed using regions-of-interest (ROI)^25,26^ and voxel-based analysis^27^. ^18^F-FES standardized uptake volume ratios (SUVR) were calculated relative to cerebellar gray matter uptake and validated against distribution volume ratios (DVR) (see Online Methods). Results are corrected for multiple comparisons, and adjusted for age, plasma E2, and modality-specific confounders.

### ROI analysis

On multivariate regression analysis, there was a significant overall effect of menopause status on ER density in *a priori* selected ER-rich ROI (pituitary, hypothalamus, nucleus accumbens, amygdala, hippocampus, striatum, thalamus, cingulate, middle and orbital frontal cortex) (multi-variable adjusted p<0.001; Table 1). In these regions, ER density generally increased in a menopause-stage dependent fashion, e.g., was highest in the post-menopausal group, intermediate in the peri-menopausal group, and lowest in the pre-menopausal group (Table 1). On post-hoc examination, the post-menopausal group exhibited higher ER density than the pre-menopausal group in all regions (multi-variable adjusted p=0.007; Table 1). Overall differences between peri-menopausal and pre-menopausal groups did not reach significance (multi-variable adjusted p=0.238; Table 1).

### Voxel-based analysis

**Figure 1:**
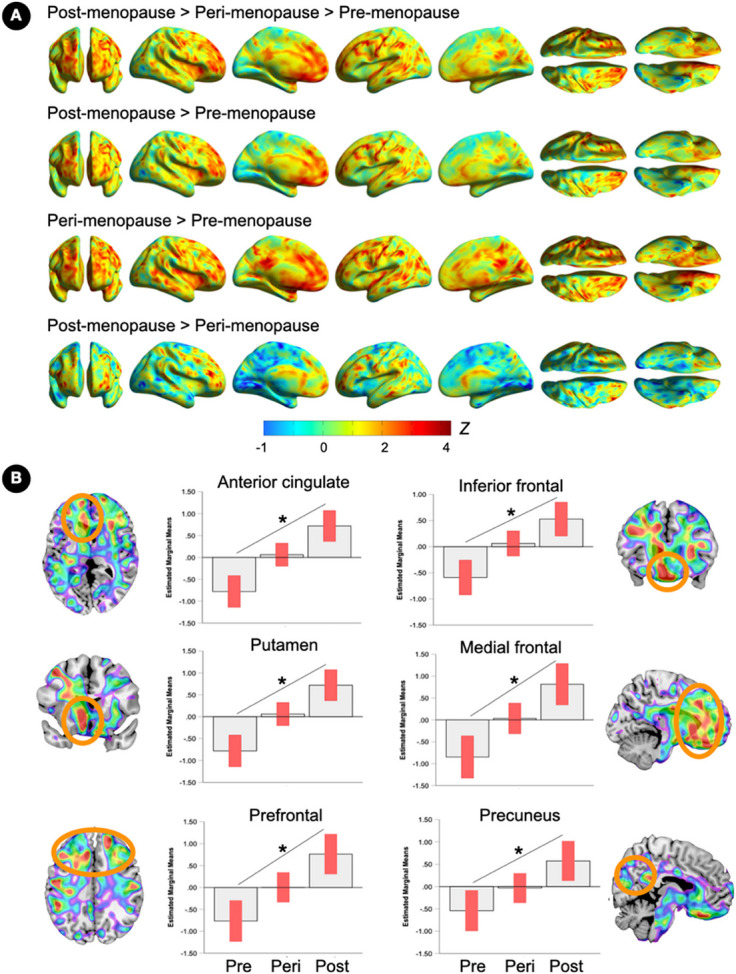
Brain estrogen receptor density by menopause stage (A) Surface maps of regional ^18^F-FES estrogen receptor (ER) density differences between pre-menopausal, peri-menopausal and post-menopausal groups (N=15 each), at family-wise error (FWE) corrected cluster-level P<0.05, adjusted by age, plasma estradiol levels and cerebellar uptake. Statistical parametric maps are represented on a spectrum color-coded scale with corresponding Z scores. Statistics are reported in Table 2. (B) Coronal, sagittal or axial slice overlays superimposed on a volumetric template MRI with corresponding plots representing estimated marginal mean signal from peak clusters. Error bars are 95% confidence intervals; *Overall effect, P<0.05. Abbreviations: Peri, peri-menopause; Post, post-menopause; Pre, pre-menopause.

Voxel-based analysis detected progressively higher ER density from pre-menopausal to post-menopausal stages, consistent with ROI results (Figure 1). As compared to the ROI approach, voxel-based analysis enhanced detection of menopause-stage effects and identified some effects of hemispheric laterality. Peak clusters of overall associations between menopause status and ER density were located in medial and middle frontal gyrus, bilaterally, as well as putamen, cingulate, orbitofrontal gyrus, and precuneus of the left hemisphere (p_FWE_≤0.042, Figure 1 and Table 2). On post-hoc analysis, the post-menopausal group exhibited higher ER density in all of these regions as compared to both pre-menopausal and peri-menopausal groups (p_FWE_≤0.026, Figure 1 and Table 2). The peri-menopausal group exhibited higher ER density in cingulate cortex, putamen, and precuneus, bilaterally, and in amygdala and inferior parietal lobule of the left hemisphere, as compared to the pre-menopausal group (p_FWE_≤0.049 Figure 1 and Table 2).

### Prediction of group membership

**Figure 2:**
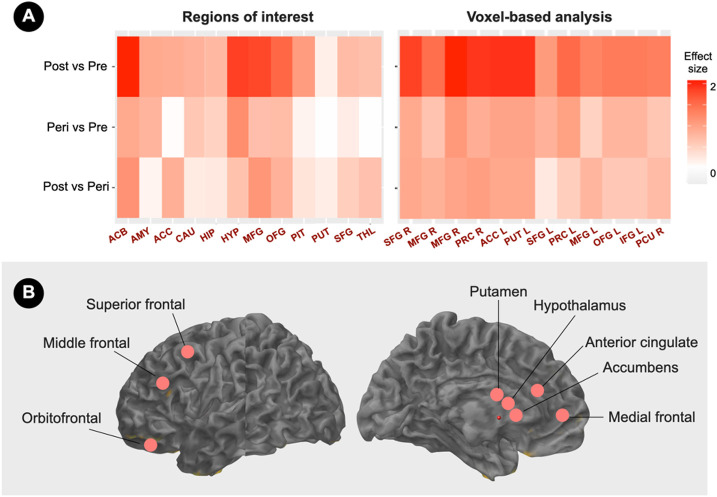
Menopause status classification by brain estrogen receptor density (A) Standardized pairwise mean estrogen receptor (ER) density differences in pre-specified regions of interest (left) and voxel-based analysis clusters (right) between menopause statuses are expressed as Cohen’s d coefficients, where d≥0.8 reflects a large effect size. (B) Schematic illustration of brain regions yielding maximum group separation, superimposed on a volumetric template MRI image. Abbreviations: ACB, accumbens; ACC, anterior cingulate; AMY, amygdala; CAU, caudate, HIP, hippocampus, HYP, hypothalamus; IFG, inferior frontal gyrus; L, left; MFG, middle frontal gyrus; OFG, orbitofrontal gyrus; Peri, peri-menopause; PCU, precuneus; PIT, pituitary; Post, post-menopause; PRC, precentral gyrus; Pre, pre-menopause; PUT, putamen; R, right; SFG, superior frontal gyrus; THAL, thalamus.

On a regional basis, ER density in hypothalamus, nucleus accumbens, medial and superior frontal cortex, anterior cingulate, and putamen yielded the largest effect size in differentiating post-menopausal from pre-menopausal participants (Cohen’s d’s ≥1.8, Figure 2). ER density in these regions resulted in 100% predictive accuracy in classifying participants as being post-menopausal or pre-menopausal (p<0.05).

### Associations of ER density with brain structure, function, and energy metabolism

To test whether ERs exert *in vivo* modulatory actions on neuronal density, cerebral perfusion and energy metabolism, we performed multimodality neuroimaging on the same participants to test for associations of ER density with (i) GMV assessed via volumetric Magnetic Resonance Imaging (MRI), (ii) CBF via Arterial Spin Labeling (ASL), and (iii) ATP production via ^31^Phosphorus Magnetic Resonance Spectroscopy (^31^P-MRS).

### ER density and Gray Matter Volume

**Figure 3:**
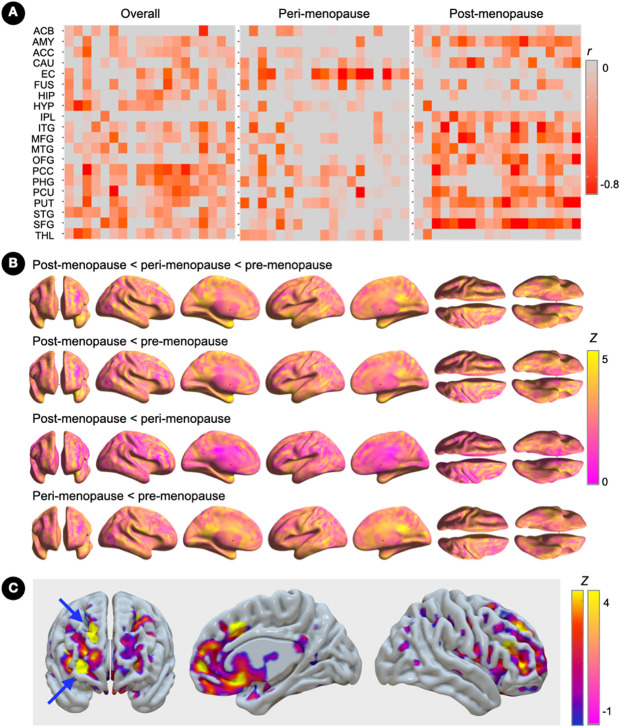
Associations of brain estrogen receptor density and gray matter volume (A) Covariate-adjusted associations between estrogen receptor (ER) density and gray matter volume (GMV) in the full regions-of-interest panel, and corresponding partial correlation *r* coefficients. (B) Surface maps of regional GMV differences between pre-menopausal, peri-menopausal and post-menopausal groups (N=15 each), at family-wise error (FWE) corrected cluster-level P<0.05, adjusted by age and total intracranial volume. Statistical parametric maps are represented on a color-coded scale with corresponding Z scores. Statistics are reported in **Supplementary Table 2.** (C) Statistical parametric maps showing overlapping effects of menopause status on ER density (blue-to-yellow color scale) and GMV (pink-yellow color scale) superimposed on the anterior, right medial and lateral views of a volume-rendered template MRI. Arrows point to peak clusters of negative correlations between modalities (bright yellow). Abbreviations: ACB, accumbens; ACC, anterior cingulate; AMY, amygdala; CAU, caudate; EC, entorhinal cortex; FUS, fusiform; HIP, hippocampus, HYP, hypothalamus; IPL, inferior parietal lobe; ITG, inferior temporal gyrus; L, left; MFG, middle frontal gyrus; MTG, middle temporal gyrus; OFG, orbitofrontal gyrus; Peri, peri-menopause; PCC, posterior cingulate cortex; PHG, parahippocampal gyrus; Post, post-menopause; Pre, pre-menopause; PUT, putamen; R, right; SFG, superior frontal gyrus; STG, superior temporal gyrus; THAL, thalamus.

Correlations between ER density and GMV in ER-rich regions exhibited a generally negative pattern, which was driven by the post-menopausal group and nearly absent in the peri-menopausal and pre-menopausal groups (Figure 3a).

As shown in Figure 3b, GMV in ER-rich regions was progressively lower from pre-menopausal to post-menopausal stages, with peak clusters localized in limbic lobe, posterior cingulate, inferior temporal and fusiform gyri (p_FWE_≤0.026; **Supplementary Table 2**). Additionally, the post-menopausal group showed lower GMV in frontal regions and putamen as compared to the peri-menopausal group (p_FWE_≤0.037; **Supplementary Table 2**). The frontal cortex exhibited the strongest negative associations between ER density and GMV, with peak clusters in superior and middle frontal gyrus of the right hemisphere (Brodmann area 9; cluster extent 21 voxels; peak coordinates x=17, y=45, z=23; Z=3.93, *r* = −.710, p_FWE_=0.004; Figure 3c).

### ER density and Cerebral Blood Flow

**Figure 4:**
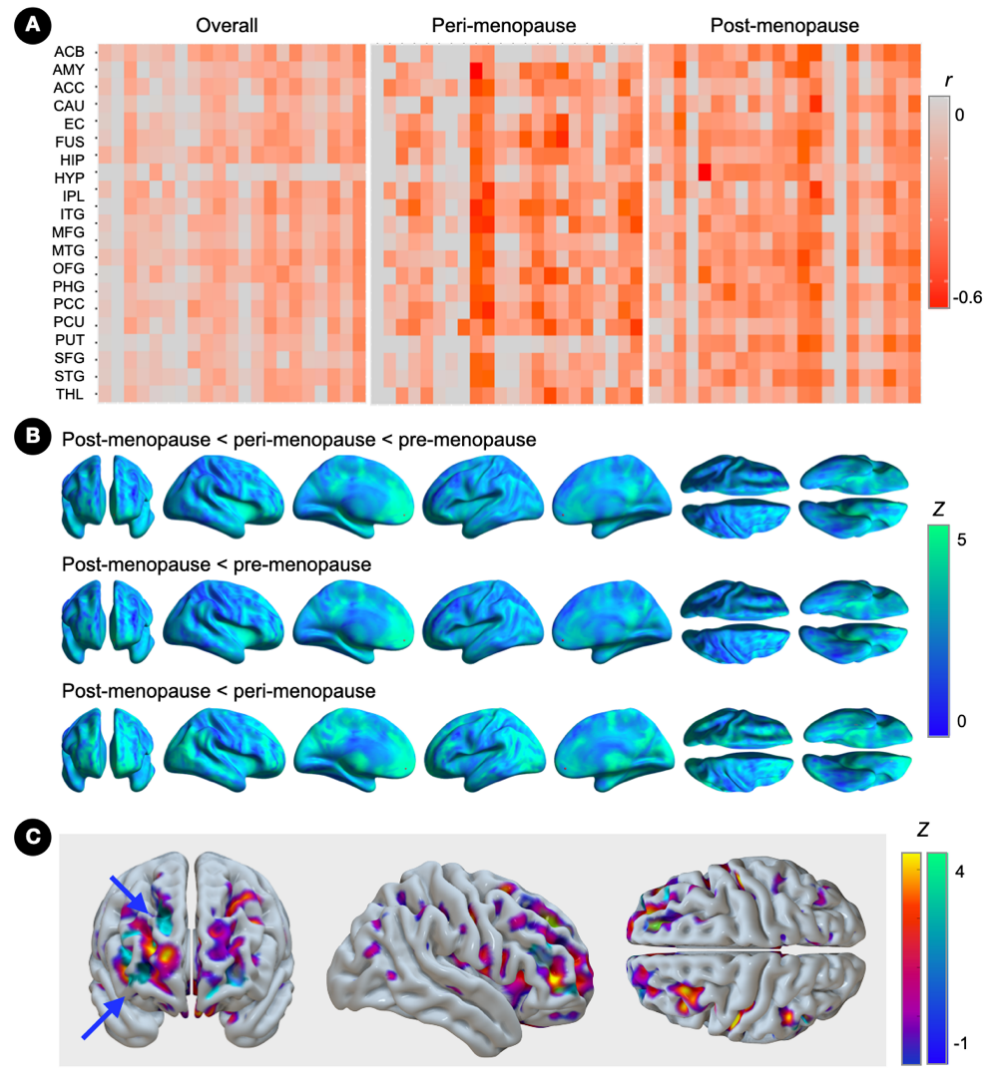
Associations of brain estrogen receptor density and cerebral blood flow (A) Covariate-adjusted associations between estrogen receptor (ER) density and cerebral blood flow (CBF) in the full regions-of-interest panel, and corresponding partial correlation *r* coefficients. (B) Surface maps of regional CBF differences between pre-menopausal (N=15), peri-menopausal and post-menopausal groups (N=14 each), at family-wise error (FWE) corrected cluster-level P<0.05, adjusted by age and global activity. Statistical parametric maps are represented on a color-coded scale with corresponding Z scores. Statistics are reported in **Supplementary Table 3.** (C) Statistical parametric maps showing overlapping effects of menopause status on ER density (blue-to-yellow color scale) and CBF (blue-green color scale) superimposed on the anterior, right medial and lateral views of a volume-rendered template MRI. Arrows point to peak clusters of negative correlations between modalities (bright blue). Abbreviations: see legend to Figure 3.

Overall correlations between ER density and ROI CBF exhibited a generally negative pattern, which were more pronounced at the post-menopausal stage (Figure 4a).

As shown in Figure 4b, CBF in ER-rich regions was progressively lower from pre-menopausal to post-menopausal stages, with peak clusters located in superior frontal and cingulate cortex, striatum and thalamus (p_FWE_≤0.037; Figure 4a and **Supplementary Table 3**). On post-hoc examination, the postmenopausal group showed lower CBF in these regions, as well as in inferior and medial frontal regions, as compared to peri-menopausal and pre-menopausal groups (p_FWE_≤0.046). The post-menopausal group also exhibited lower CBF in hippocampus as compared to the pre-menopausal group (p_FWE_=0.044; Figure 4a and **Supplementary Table 3**). The frontal cortex exhibited the strongest negative associations between ER density and CBF, with peak clusters in middle and superior frontal gyrus of the right hemisphere (Brodmann area 46/10; cluster extent 22 voxels; peak coordinates x=37, y=35, z=12; Z=3.41, *r* = −0.423, p_FWE_=0.002; Figure 4c).

### ER density and ATP production

**Figure 5:**
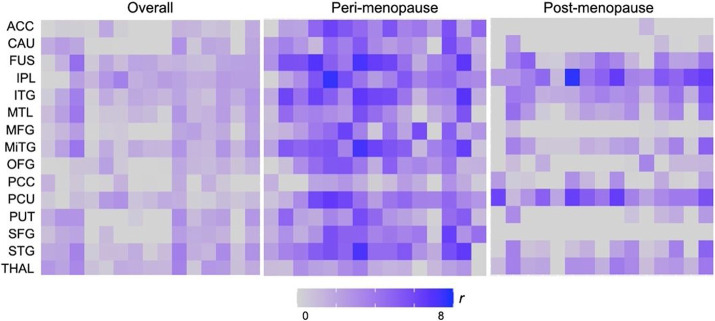
Associations of brain estrogen receptor density and mitochondrial ATP production Covariate-adjusted associations between estrogen receptor (ER) density and phosphocreatine to ATP (PCr/ATP) measures in the full regions-of-interest panel, and corresponding partial correlation coefficients overall (N=42), and separately for peri-menopausal (N=14) and post-menopausal groups (N=13). Abbreviations: ACC, anterior cingulate; CAU, caudate; FUS, fusiform; IPL, inferior parietal lobe; ITG, inferior temporal gyrus; L, left; MFG, middle frontal gyrus; MTG, middle temporal gyrus; MTL, medial temporal lobe; OFG, orbitofrontal gyrus; Peri, peri-menopause; PCC, posterior cingulate cortex; Post, post-menopause; Pre, pre-menopause; PUT, putamen; R, right; SFG, superior frontal gyrus; STG, superior temporal gyrus; THAL, thalamus.

As shown in Figure 5, overall correlations between ER density and the ratio of phosphocreatine to ATP (PCr/ATP), reflecting higher ATP utilization, exhibited a generally positive pattern.On examination of each menopausal group, these correlations were more pronounced at the peri-menopausal stage and less pronounced post-menopause (Figure 5). In the peri-menopausal group, the strongest positive regional associations were in fusiform, inferior parietal lobe, and thalamus (r≥0.395, p<0.05). In the post-menopausal group, the strongest effects were in fusiform gyrus and putamen (r≥0.256, p<0.05).

### Associations of ER density with Menopause Symptoms

We then tested for associations between ER density and self-reported changes in thermoregulation (vasomotor symptoms), mood, sleep, cognition, and libido.

**Figure 6:**
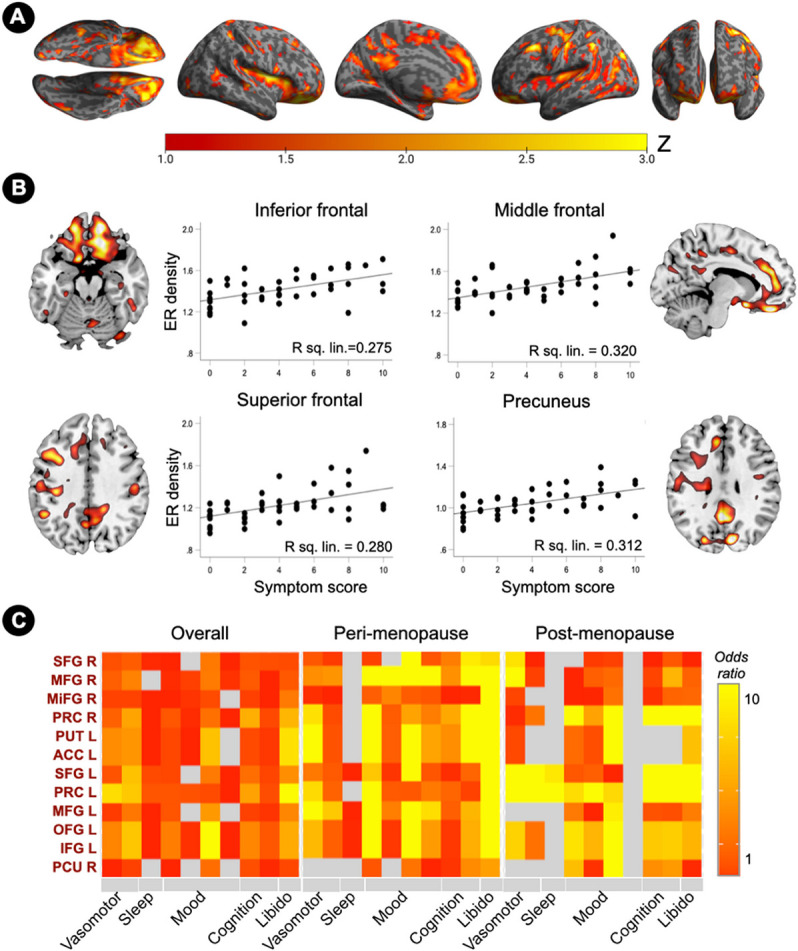
Associations of brain estrogen receptor density and menopause symptoms (A) Surface maps of regional associations between estrogen receptor (ER) density and menopause symptom scores, at cluster-level P_FWE_<0.05, adjusted by age and cerebellar uptake. Statistical parametric maps are represented on a color-coded scale with corresponding Z scores. Statistics are reported in **Supplementary Table 4**. (B) Sagittal or axial slice overlays superimposed on a volumetric template MRI and corresponding correlation plots of standardized uptake value ratios (SUVR) from peak clusters and menopause symptoms scores. (C) ER density predicts presence of menopause symptom clusters. Odds ratios are estimated for the entire sample (N=45) and separately for peri-menopausal and post-menopausal groups (N=15 each), and displayed on a color-coded scale. Abbreviations: see legend to Figure 2.

ER density was positively associated with a composite menopause symptom severity score in inferior frontal gyrus and precuneus, bilaterally; superior frontal gyrus, anterior cingulate and precentral gyrus of the left hemisphere; and middle and orbitofrontal gyrus of the right hemisphere (p_FWE_≤0.050; Figure 6a,b and **Supplementary Table 4**).

In examination of menopause symptom clusters, associations between ER density in voxel-based clusters showing menopause effects and symptom presence were more pronounced in the peri-menopausal group, and attenuated post-menopause (Figure 6c). In the peri-menopausal group, ER density significantly predicted changes inlibido (odds ratios, OR=9.5, 95% confidence interval, 4.2-10), mood changes (OR=7.9, 95% CI, 0.7-10), difficulty concentrating (OR=6.5, 95% CI, 4.1-10), and hot flashes (OR=6.1, 95% CI, 0.39-10) (Figure 6c). In the post-menopausal group, ER density in the same clusters also predicted changes inlibido (OR=5.8, 95% CI, 0.2-10), tearfulness (OR=7.3, 95% CI, 0.43-10), difficulty concentrating (OR=5.5, 95% CI, 0.2-10), and hot flashes (OR=5.2, 95% CI, 0.2-10), as well as memory difficulties (OR=4.8, 95% CI, 0.6-10) (Figure 6c).

Similar results were found using ROI measures, with anterior brain regions, chiefly frontal cortex, anterior cingulate and basal ganglia, predicting most menopause symptoms at the peri-menopausal stage, and less so at the post-menopausal stage (**Supplementary Table 5**). Additionally, ER density in hypothalamus and amygdala predicted presence of peri-menopausal night sweats (OR=10, 95% CI, 0.3-10, and 8.7, 95% CI, 0.1-10, respectively, **Supplementary Table 5**). ER density in hypothalamus predicted presence of post-menopausal hot flashes and night sweats (OR=10 and 5.2, respectively, 95% CI, 0.2-10), as well as sleep disturbances (OR= 5.9, 95% CI, 0.2-10).

## Discussion

This *in vivo* multi-modality imaging study demonstrates progressively higher brain ER density over the menopause transition, with associated alterations in brain volume, perfusion, and energy metabolism. Results demonstrate high anatomical overlap with estrogen-regulated brain networks involved in both reproductive function and higher-order cognitive functions, and were independent of chronological age and plasma estradiol (E2) levels. Elevations in ER density were highly consistent, correctly classifying all post-menopausal women. Finally, ER density predicted presence of menopause symptoms, chiefly changes in libido, mood, cognition, and vasomotor symptoms.

For decades, the classic view of estrogen action in brain was confined to regulation of ovulation and female reproductive behavior^1,2^. Later studies challenged this paradigm, identifying E2 as the “master regulator” of neurological function in female brain due to its broad impact on multiple neural processes^2 3^. Further advances led to discovery that E2 actions are mediated by specialized brain ERs, including classical ER alpha (ERα) and beta (ERβ), which are found in neurons, glial cells, astrocytes; and G protein-coupled estrogen receptor 1 (GPER1) mainly located in plasma membranes^1,2^. ERs modulate synaptic plasticity, adult neurogenesis, and DNA repair^4^, as well as expression of a wide variety of genes linked to lipid metabolism, vasodilatation, synaptic potentiation, and myelination^1,2^. Major projection neurons such as cholinergic, serotonergic, and dopaminergic systems are also responsive to ER activity^5,6^, further highlighting the importance of ER-mediated activity for brain health. However, our knowledge of ER expression in the living human brain is very limited.

PET imaging with ER ligands is the only technique currently available to assess ER expression *in vivo*. ^18^F-FES is the most utilized ER tracer thanks to extensive pharmacological validation of its binding properties against biopsy material assayed by *in vitro* radioligand binding and immunohistochemistry^17,18^. As ^18^F-FES exhibits higher affinity for ERα than other ERs^19^, the signal originates mostly from ERα-expressing areas.

Brain ^18^F-FES PET studies are however scarce, and mostly in animals. Preclinical studies have been instrumental in demonstrating high tracer binding in regions with known ERα expression, chiefly pituitary and hypothalamus^19-21^, as well as striatum, limbic lobe, and cortex^22^. In female rats, oophorectomy provoked a marked increase in pituitary and hypothalamic ER density relative to non-oophorectomized controls^20,21^, as well as smaller increases in amygdala and frontal cortex^22^. This indicates that E2 declines following oophorectomy (surgical menopause) prompt an increase in ER expression, which could be reduced by pre-surgical administration of estradiol.^20 22^

Whether similar mechanisms are active in human endocrine aging is unknown. Currently, the only brain ^18^F-FES study in women was conducted on *de novo* breast cancer patients, showing a trend toward higher pituitary uptake in post-menopausal as compared to pre-menopausal patients^21^. The pre-menopausal group was substantially smaller than the post-menopausal group, as expected in a breast cancer cohort, and PET imaging in the pre-menopausal group was not standardized to the menstrual cycle^21^, which may have reduced power to detect group differences. Additionally, delineation of the pituitary region was based on tracer uptake rather than MRI-guided tracing, and pituitary uptake was normalized to frontal cortex^21^, a known ER site^28-31^, which may have confounded detection of menopausal effects.

The present brain ^18^F-FES PET study examined a prospective cohort of healthy midlife women ages 40-65 years, divided into three size-matched groups based on menopausal status. All post-menopausal women had undergone menopause spontaneously, and pre-menopausal participants were scanned around midcycle. We used state-of-the-art ROI and voxel-based analysis to examine ^18^F-FES data normalized to a statistically-validated cerebellar reference region developed via supervised clustering algorithms (see Online Methods and below). Using these procedures, our results show a pattern of brain ER distribution which maps onto estrogen-regulated neural systems^3^, and is consistent with preclinical *ex vivo*^28-31^ and *in vivo* work^19-21^. Consistent with the above breast cancer study^21^, the pituitary gland showed the strongest tracer uptake, which was highest post-menopause, intermediate in peri-menopause, and lowest pre-menopause. Hypothalamus as well as other ERα-abundant brain regions, mainly thalamus, striatum, midbrain, cingulate, frontal cortex, and medial temporal lobe, also exhibited progressively higher ER density from pre-to post-menopausal stages. These effects were independent of age and highly consistent, correctly identifying all post-menopausal women vs. pre-menopausal controls. Additionally, by focusing on spontaneous menopause, we were able to detect alterations in ER density at the peri-menopausal stage, chiefly in frontal cortex, cingulate, amygdala and putamen. Overall, these findings provide novel evidence that female endocrine aging is associated with progressively higher ER expression in E2-regulated areas involved in various higher-cognitive functions including mood, memory, stress, pain, and fine motor skills^1,2^, with onset in peri-menopause.

Higher ^18^F-FES binding post-menopause may be due to either the near-absence of endogenous E2 competing for receptor occupancy, or to an estrogen depletion-induced positive feedback loop triggering ER over-expression as a compensatory response aimed at preserving neural function in E2-reliant regions. While dilution effects cannot be fully excluded, several lines of evidence support presence of a compensatory mechanism. First, the surgical histology studies described above provide an important part of the basis for concluding that increased tracer uptake post-menopause reflects increased ER density, rather than another possible explanation such as less competition from available E2^19-21^. Secondly, studies in rodents have shown that brain ER expression is in part negatively associated with E2 levels during the menstrual cycle^32^, which supports evidence for increased post-menopausal expression once E2 levels are permanently low. Third, in terms of tracer kinetics, preclinical studies have shown that delivery of ^18^F-FES to the brain may be affected by circulating E2 whereas its binding to ERs is less or not impacted^20^. Hormonal differences between female rats and women notwithstanding, studies of breast cancer patients also reported mild or non-significant effects of circulating E2 on ^18^F-FES uptake in peripheral tissues^33^. In the present study, tracer uptake did not significantly correlate with plasma E2, and group differences in tracer uptake remained significant adjusting by E2 levels.

While E2 can regulate its own receptor gene expression in the adult brain^33^, other factors control ERα mRNA in addition to circulating E2^34^. For instance, E2 is mainly produced in the ovary, but it is also locally synthesized in brain^1,2^. Brain steroidogenesis is regulated independently of peripheral steroidogenesis, and plasma steroid levels do not directly reflect brain steroid levels^4^, which may have contributed to our findings. Further, after oophorectomy in female rats, the brain adapts the levels of steroid synthesis as a compensatory adaptation of brain steroidogenesis in response to gonadal steroid deprivation^35^. Although aromatase activity is reduced with aging, the brain retains the ability to synthesize E2 locally under conditions of neurological stress^4^. In response to brain injury and stroke, endogenous E2 synthesis and ER expression are both upregulated in neurons, and *de novo* synthesis is induced in astrocytes, independent of ovarian E2 production^4^. It is possible that neurological stress during the menopause transition might trigger similar responses. Additionally, estrone (E3, the most prevalent type of estrogen post-menopause) can both bind to ERα and be converted into E2. This may further justify the need for an increase in ER density. Overall, elevations in brain ER density independent of menopause-induced plasma E2 fluctuations are in line with a compensatory neurophysiological response to loss of ovarian E2.

Our correlational analysis of multimodality imaging outcomes also supports the notion that ER overexpression may occur as a compensatory reaction. In fact, higher ER density was generally associated with lower GMV and CBF in ER-rich regions, with peak effects noted in frontal cortex. These associations were more pronounced at the post-menopausal stage, consistent with observations that E2 declines prompt decreases in synaptic density and in CBF through its actions on ERs^1,2,5,6^. Previous imaging studies of midlife women also report lower frontal GMV and functional activity in post-menopausal and peri-menopausal women as compared to pre-menopausal controls and age-controlled men.^10-13^

On the other hand, ER density was associated with higher ATP production in estrogen-dependent regions - - an effect that was more pronounced at the peri-menopausal stage and attenuated post-menopause. ERs are found in mitochondria, which are a site of both E2 synthesis and estrogen-inducible neuroprotective actions^36^. Acting on ERs, E2 regulates cerebral glucose metabolism (CMRglc), the tricarboxylic acid cycle-coupled oxidative phosphorylation and ATP generation in mitochondria of neurons and glial cells^36^. In mechanistic analyses, ER protein synthesis is unchanged during peri-menopause, whereas transcription mechanisms are impaired, reflecting a disassembly between ER and cerebral metabolic systems^3,37,38^. The resulting loss of estrogenic-controlled glucose metabolism triggers a compensatory bioenergetic adaptive response to utilize lipids as an auxiliary fuel to restore ATP^3,37,38^. As midlife women exhibit declining CMRglc during the menopause transition^10-13^., present results of greater ER-related ATP utilization are consistent with a compensatory reaction to altered cerebral bioenergetic processes, as well as to declines in GMV and perfusion. The raise in ATP may also reflect the energetic cost of sustaining ER over-expression, warranting further investigation.

Another novel finding was the association of ER density with presence of menopausal symptoms of neurological origin, chiefly vasomotor symptoms, mood changes, low sex drive, and ‘brain fog’. Declines in circulating sex hormones, chiefly E2, are assumed to account for these changes^8^, thought direct evidence of this is lacking. Present results indicate that increasing ER levels in estrogen-regulated regions are associated with an over 5-fold increase in the odds of experiencing the above neurological symptoms. These associations were stronger at the perimenopausal stage and attenuated post-menopause, consistent with clinical observations that menopausal symptoms tend to subside a few years after the final menstrual period^8^.

Moreover, mounting evidence identifies deprivation of estrogenic effects following menopause as a key biological mechanism underlying women’s greater vulnerability to brain conditions such as mood disorders, AD, and stroke^39,40^. Because ERs mediate estrogen actions, and ERs may themselves participate in disease development, ER-PET imaging represents an essential advance in our ability to develop and test estrogen-based therapies for prevention and treatment of many brain disorders.

Estrogen is the recommended short-term treatment for menopausal symptoms such as hot flashes. Menopause hormone therapy (HT) also holds promise for attenuation of neuronal injury and cell death resulting from neurodegenerative insults^9,40,41^. However clinical studies have produced mixed results, as observational studies generally report decreased neurological risk in women taking HT for menopausal symptoms as compared with non-users, whereas clinical trials of on older post-menopausal women have produced negative or null results^15,41,42^. Experts argue that HT is best started early in the course of menopause, as therapy has no clear beneficial effects once neurological disease is clearly established^43^.

Therefore, as women approach midlife, there seems to be a critical window of opportunity not only to detect signs of neurological risk but to then intercede with strategies to reduce or prevent that risk by ameliorating estrogen levels^44^. Long-term E2 deprivation has been shown to lead to degradation of ERα in some regions^45^. Present neuroimaging findings indicate that the brain is undergoing estrogenic and biochemical adjustments starting in perimenopause, which are still active in early post-menopausal women. These results help hone in on the window of opportunity for therapeutical intervention, and provide biological markers of neurological vulnerability for future clinical trials of estrogen therapy.

## Strengths And Limitations

To our knowledge, this is the first *in vivo* brain imaging study to investigate ER modulation by neuroendocrine aging in humans. We focused on carefully screened, healthy midlife women ages 40-65 years, with complete clinical and cognitive exams, laboratory tests, menopause assessments, and brain imaging. Our extensive exclusion criteria ensured absence of confounding factors such as cancer, oophorectomy / hysterectomy, and HT use. From a methodological perspective, we examined women at different menopausal stages, paired with age correction procedures, as a natural experiment of estrogenic decline. We used a combination of state-of-the-art ROI and voxel-based analysis while taking a translational approach to ensure that our results were both statistically and biologically valid. Results were significant after a stringent correction for multiple comparisons and multi-variable correction for age, modality-specific confounders, and plasma E2 levels immediately prior to imaging. We chose a cross-sectional design because the timing of menopause is highly variable, with a median age at menopause of 51 years and distribution 40-58 years^8^. Longitudinal studies may require 10-15 years of follow-ups to capture changes in brain ERs in the same women. While oophorectomy ideally reduces follow-up times, the procedure yields different neurophysiological outcomes^7,8^. Nonetheless, given the cross-sectional and observational nature of this study, a temporal and causal relationships between exposures and outcomes cannot be unequivocally established. Longitudinal studies are warranted to characterize the temporal trajectories of ER changes in relation to endocrine aging, and to test for differences between induced and spontaneous menopause.

While the perimenopausal group exhibited slightly lower cognitive scores than the other groups, cognitive performance did not differ by menopause status. This is consistent with clinical observations that, while self-reports of poor memory and concentration are common in women of menopausal age, especially in perimenopause, menopause itself isn’t associated with objective cognitive deficits^8^. However, lack of variability in cognitive measures prevented us from examining associations between ER density and cognition. Overall, we caution that present results are obtained from small samples of carefully screened research participants with at least 12 years of education. Replication in community-based populations with diverse educational, racial or socio-economical background is warranted.

Kinetic modeling with absolute quantification remains the gold-standard for PET neuroreceptor studies. Kinetic ^18^F-FES studies in animals report high specific binding in pituitary and hypothalamus^19-22^, which was confirmed by preliminary reports in humans^23,24^. Absolute quantification is however invasive due to the need for continuous arterial blood sampling, while also being subject to error-prone metabolite analysis^46^. Herein, we used graphic reference-tissue Logan plots^47^ to derive ^18^F-FES distribution volume ratios (DVR), and then used statistical procedures to determine that SUVR yielded comparable results to DVR in target regions (Online Methods). SUVR are preferred for clinical studies because of shorter scan duration, no need for arterial cannulation or dynamic imaging, and computational simplicity. A major limitation of the SUVR approach is that it can overestimate receptor binding relative to DVR. In animal studies, oophorectomy provoked a 1.7-fold increase in ER binding in pituitary and hypothalamus as compared to the control group^20,21^. In our study, the post-menopausal group exhibited 12% and 11% higher SUVR than the pre-menopausal group in those regions, respectively, which seems physiologically plausible for women undergoing spontaneous menopause. Furthermore, overestimation would affect all menopausal groups equally.

Another limitation of SUVR relies on the choice of a suitable reference region. As described in Online Methods, our reference ROI was selected based on preclinical *ex vivo* and *in vivo* evidence that cerebellar crus II gray matter generally does not express, or minimally expresses ERα^28-31^. The ROI was further optimized by means of supervised clustering algorithms (SCA) to ensure that tracer uptake was both low and invariant by exposure (e.g. menopause status), which is a key prerequisite for normalization^48^. SCA methods are commonly used to extract pseudo-reference regions for non-invasive quantification of PET tracers with low intracerebral uptake, such as activated microglia TSPO (translocator protein) ligands^49^. Nonetheless, it is possible that we may have underestimated ER density in the cerebellar ROI. This would however conservatively reduce power in detecting ER density effects. Given the high anatomical agreement between the observed brain ER distribution and preclinical literature; the robust effect size resulting from group comparisons; and the biological plausibility of correlational patterns with additional brain biomarkers, we attribute our results to cerebral ER expression in response to ovarian E2 declines in midlife female endocrine aging. More studies using fully kinetic modeling with absolute quantification are needed to replicate these findings.

Finally, ^18^F-FES signal is limited by non-specific binding. Novel ER ligands with lower nonspecific signal and better contrast than ^18^F-FES PET are being developed, such as 4-fluoro-11β-methoxy-16α-^18^F-fluoroestradiol (^18^F-4FMFES) ^21^. PET tracers with high selectivity for ERβ, such as 2-[^18^F]-fluoro-6-(6-hydroxynaphthalen-2-yl)pyridin-3-ol (^18^F-FHNP), as well as progesterone receptor (PRs) ligands such as ^18^F-fluoro-furanyl-norprogesterone (^18^F-FFNP) are of considerable interest for neuroscience.

Overall, because ERs mediate estrogen actions, and changes in ER expression themselves participate in cognitive function, mental health and disease development, ER-PET imaging represents an essential advance in our understanding of sex hormones’ impact in brain -- while also opening the possibility of using ER ligands to promote neuroprotection, and predicting the efficacy of estrogen treatment in clinical trials and on an individual basis.

## Conclusion

These data provide novel evidence for *invivo* ER-mediated estrogenic effects on brain structure, perfusion and energy production, with clinical implications for midlife women. Findings provide a neurobiological substrate to the neurological vulnerability reported in menopausal women, and identify a window of opportunity for preventative strategies.

## Online Methods

### Participants

This is a natural history, non-interventional study of 45 consecutive clinically and cognitively normal midlife women at different endocrine stages, including equal proportions of pre-menopausal (standardized to midcycle), peri-menopausal, and post-menopausal participants. Participants were recruited at Weill Cornell Medicine (WCM) between 2021-2022 from multiple community sources, including individuals interested in research participation, family members and caregivers of impaired patients at our institution, and by word of mouth^10-13^.

All participants gave written informed consent to participate in this ^18^F-fluoroestradiol (^18^F-FES) positron emission tomography (PET) study,which was approved by the WCM Institutional Review Board. Use of ^18^F-FES was carried out under WCM Radioactive Drug Research Committee and National Cancer Institute (NCI) Investigational New Drug (IND) #146703 approval.

Participants were 40-65 year-old women with ≥12 years of education and a diagnosis of normal cognition per physician’s assessment, with Montreal Cognitive Assessment (MoCA) scores ≥26 and cognitive test performance within normative values for age and education^10-13,12,50,51^. Pre-established exclusion criteria included: (i) any significant neurological disease, such as dementia, normal pressure hydrocephalus, brain tumor, progressive supranuclear palsy, seizure disorder, subdural hematoma, multiple sclerosis, or history of significant head trauma followed by persistent neurologic deficits or known structural brain abnormalities; (ii) any significant psychiatric disease, such as major depression, bipolar disorder, schizophrenia, or psychotic features; (iii) T2 and/or FLAIR MRI brain scan evidence of infarction, lacunes or demyelination disease; (iii) systemic illnesses, unstable medical conditions or major medical complications such as treatment for neoplastic disease, unmanaged cardiovascular disease, diabetes, renal or liver disorder; (iv) history of drug or alcohol dependence; (v) current use of psychoactive medications (e.g. benzodiazepines, cholinesterase inhibitors, psychostimulants, etc.) or investigational agents; (vi) contraindications to MRI or PET imaging. Additional exclusion criteria included: (vii) history of oophorectomy or hysterectomy; (viii) use of hormonal therapy; (ix) active pregnancy.

All participants underwent clinical examinations including medical history, neurological exams, neuropsychological testing, blood analysis including genetics and sex steroid hormones, multi-modal MRI and ^18^F-FES PET imaging. The patients’ sex was determined by self-report.

Herein, we capitalized on the menopause transition as a natural experiment of estradiol decline. Participants were enrolled into three size-matched groups according to menopausal status based on the Stages of Reproductive Aging Workshop (STRAW)^52^ with hormone laboratory assessments as supportive criteria (pre-menopause: no change in menstrual regularity in the past 12 months; peri-menopause: no menses in the past 3–11 months; post-menopause: no menses for the past ≥12 months)^52^. Participants were therefore not randomly assigned to groups. Sex steroid hormone levels, including estradiol (E2), were measured by a commercial laboratory (Boston Heart Diagnostics, Framingham, MA).

All participants were asked to report the date of their last two menstrual periods for diagnostic purposes. PET studies of pre-menopausal participants were scheduled to coincide with the next nearest midcycle, when plasma E2 levels are highest. Cycle irregularities in peri-menopausal women prevented scheduling their PET scans according to a specific menstrual cycle phase. However, plasma E2 levels were included as a covariate in statistical analysis. Blood samples were taken on the day of the PET study for all participants except two who did the blood draw the day prior, and one that was done a week later. To test whether tracer binding at midcycle was impacted by tracer competition with endogenous E2, we included E2 as a covariate, which only enhanced differences in ER density between menopausal groups. Results remained unchanged excluding E2 data from the last participant.

Our final study cohort included 45 consecutive participants, divided into three size-matched groups of 15 participants each according to menopause status: 15 pre-menopausal, 15 peri-menopausal, and 15 post-menopausal participants. Three of the original 45 participants had to be excluded due to conditions encountered in the MRI scan (demyelination; 1 pre-menopausal and 1 post-menopausal participant) or positive pregnancy test (1 pre-menopausal participant). As such, 3 additional participants were enrolled in those respective groups. Participant characteristics of the final sample are shown in **Supplementary Table 1.** Unless explicitly stated otherwise (in case of analyses including measures only available for a subset of the participants), these represent the sample sizes used in analysis.

There were no differences in demographic measures between menopausal groups, except for an expected age difference between the pre-menopausal and post-menopausal groups (**Supplementary Table 1**), which was addressed according to published protocols^12,53^. Briefly, we used box plots and frequency diagrams to ensure that we had sufficient age overlap among different menopause statuses, which enabled us to test for effects of endocrine aging separately from chronological aging. Age was then set as a covariate in all analyses.

All participants completed menopause questionnaires, including rating scales for symptom clusters including presence of vasomotor symptoms, changes in mood, sleep, libido, and cognition^10-13^. A composite menopause symptom score was calculated as the sum of individual scores, with higher values reflecting more symptoms. These scores were higher in the post-menopausal (4.5±3.18) and peri-menopausal groups (5.3±3.1) as compared to the pre-menopausal group (1.7±1.3; p’s≤0.010). Menopause symptom scores and symptom presence were examined as correlational outcomes.

### Cognitive Testing

Participants underwent a cognitive testing battery assessing general cognitive functioning, memory [Rey Auditory Verbal Learning Test (RAVLT) total, immediate and delayed recall; Wechsler Memory Scale logical memory immediate and delayed recall], executive function [Trail Making Test (TMT)-B; FAS], and language [animal naming; WAIS object naming]^10-13,50,51^. We then calculated a composite verbal memory score by z-scoring both delayed recall tests and averaging across measures. Additionally, a global cognition score was obtained by z-scoring each of the remaining tests and averaging within and across domains. TMT-B scores were first inverted by multiplying by −1. Descriptive assessment of cognitive scores across menopause statuses showed no significant differences, adjusting by age and years of education (p’s>0.69; **Supplementary table 1**). As cognitive scores differed by less than 1 standard deviation between menopause groups, the variance in these measures was deemed too small to enable meaningful assessment of correlations with ^18^F-FES data.

### Brain Imaging

#### Acquisition

All participants received MRI and PET scans following standardized protocols^10-13^. Scans were performed on consecutive days, except for 9 participants who completed FES an average of 0.8±1.9 months before or after MRI. Adjusting by time between scans as a covariate did not significantly impact correlational results.

All MR sequences were acquired on a 3.0 T MR750 Discovery scanner (General Electric, Waukesha, WI) using a 32-channel head coil in a single imaging session, including:

##### Volumetric MRI:

3D volumetric T_1_-weighted MRI [BRAVO; 1x1x1 mm resolution, 8.2 ms repetition time (TR), 3.2 ms echo time (TE), 12° flip angle, 25.6 cm field of view (FOV), 256x256 matrix with ARC acceleration].

##### Arterial Spin Labeling (ASL):

acquired using a pseudo-continuous technique [4851 ms TR, 10.6 ms TE, 4 averages, 24 cm FOV, 2.0x2.0x3.8 mm resolution] to estimate cerebral blood flow (CBF) from arterial blood water^54^. One post-menopausal participant did not complete the scan due to technical issues.

##### ^31^Phosphorus Magnetic Resonance Spectroscopy (^31^P-MRS):

acquired on the same scanner using a dual tuned ^31^P/^1^H quadrature head coil (Clinical MR Solutions, Brookfield, WI). Prior to MRS scanning, shimming was performed using a ^1^H single voxel technique placed over the entire brain avoiding the air-tissue interfaces. Multiple 2D slices were acquired resulting in an 8x8x8 grid with a 24 cm FOV [2048 points, 5000 Hz sweep width, 2000 ms TR, 2 averages, 55° flip angle at 51.3 MHz] in the sagittal plane. An 8 slice sagittal T_1_-Fluid Attenuated Inversion Recovery sequence [FLAIR; 2200 ms TR, 12 ms TE, 780 ms inversion time (TI), 24 cm FOV, 0.94x0.94 mm] was acquired with a 5 mm slice thickness at exactly the same position as the center of each ^31^P MRS slice for reference. The central 4 slices were coregistered to MRI by using the 8-slice concordant image set acquired at the time of MRS. Two peri-menopausal and one post-menopausal participants did not complete the ^31^P-MRS procedure due to technical issues.

##### ^18^F-Fluoroestradiol PET imaging.

16α-[^18^F]fluoro-17β-estradiol (^18^F-FES) was prepared by the WCM PET Radiochemistry Group using established methods for synthesis and quality assurance^55,56^ [https://imaging.cancer.gov/programs_resources/cancer-tracer-synthesis-resources/FES_documentation.htm]. ^18^F-FES scans were acquired using a Siemens BioGraph mCT 64-slice PET/CT scanner [70 cm transverse FOV, 16.2 cm axial FOV] operating in 3D mode. All scans were performed after a 4-hour fasting to decrease biliary uptake. One hour before PET imaging, an antecubital venous catheter was positioned for tracer injection. No arterial blood sampling was performed. Participants lied down on the scanner bed with eyes closed and ears unplugged, in the quiet and dimly lit scan room. Following a low-dose CT scan, a dose of approximately 6 mCi (222 MBq) of ^18^F-FES was infused intravenously in a volume of 20 mL isotonic phosphate buffered saline containing less than 15% of ethanol by volume over 2 minutes. Dynamic imaging was performed for 90 minutes, and consisting of 30 frames: 4x15, 4x30, 3x60, 2x120, 5x240, 12x300 sec. All images were corrected for attenuation, scatter and radioactive decay.

### ^18^F-FES analysis

#### Regions of Interest

##### Target regions.

While ERs are widely expressed throughout the brain, their density varies by isoform and region^1,2^. As ^18^F-FES selectively binds ERα, and tracer uptake in white matter is confounded by non-specific binding^23,24^, we focused on predominantly gray matter regions with high ERα expression. These included pituitary, hypothalamus, thalamus, hippocampus, amygdala, midbrain, striatum, cingulate, medial and orbitofrontal cortex^28-31^. These regions were carried into hypothesis testing. Additionally, we examined regions with lower ERα expression but known E2-related effects: superior, middle, inferior and precentral frontal cortex; precuneus; entorhinal cortex, parahippocampal gyrus; inferior, middle and superior temporal gyri; fusiform; superior and inferior parietal lobule^28-31^.

##### Reference region.

We chose the cerebellum as the anatomical reference region based on evidence that it is generally void of ERα^17,20-22^. We then developed a probabilistic cluster-based cerebellar ROI, using the following procedures, detailed in **Supplementary Figure 1:** (i) to be maximally conservative, as ERβ and GPER-1 are expressed in the innermost portion of cerebellar white matter and adjacent gray matter (corresponding to human middle cerebellar peduncle, culmen, arbor vitae, dentate nucleus, and medullary cortex)^17,20-22^, the cerebellar ROI was restricted to the outermost portion of cerebellar crus II gray matter, which is generally free of ERs; (ii) voxel-based machine learning with intensive iterative data resampling implemented in NPAIRS (nonparametric prediction, activation, influence, and reproducibility resampling)^57^ identified the inferior portion of cerebellar crus II as showing invariant tracer uptake across menopause classes, which is a pre-requisite for data normalization^48^. The final cerebellar ROI is illustrated in **Supplementary Figure 1.**

#### Kinetic modeling and simplified reference-tissue analysis

^18^F-FES data were examined as distribution volume ratios (DVR) and standardized uptake value ratios (SUVR) using ROIs based upon anatomical labeling atlas (AAL3)^58^ ROIs restricted to grey matter using a smoothed gray segment image from each participant’s volumetric MRI. The pituitary ROI was manually delineated on the coregistered anatomical MRI by two expert raters (A.P. and L.M.) using a 1 cm radius sphere, according to published criteria^59^. ROI placement, thresholding, and sampling were conducted using PMOD v4.1 (PMOD Technologies).

##### Distribution volume ratios.

ROIs were applied to motion-corrected dynamic PET images to obtain regional time activity curves (TAC, mCi/mL) of tissue radioactivity concentration across all slices sampled. Graphic reference-tissue Logan plots^47^ were used to estimate ^18^F-FES DVR (1+BP binding potential) as the concentration of the radioligand in each target region relative to tracer concentration in the cerebellar reference ROI, as implemented in PMOD 4.1.

##### Standardized uptake value ratios.

For comparisons with previous work^21^, and to assess whether brain ER expression could be estimated from a single, late-scan static PET image, summed PET images corresponding to 30-90, 30-60, and 50-70 minutes of ^18^F-FES data were converted to standardized uptake values [SUV = activity (Bq/g)/[injected activity (Bq)/body weight (g)]. SUV_Max_ were extracted from pituitary ROI, and average SUV (SUV_Mean_) from other regions, and normalized to cerebellar cortex ROI to obtain SUVR for each timeframe. SUVR are more suitable for clinical application.

##### Reliability analysis.

DVR and SUVR values were compared in SPSS v.28 (IBM) using Intra-class Correlation Coefficients (ICC) with Cronbach’s Alpha as reporting criterion, p<0.05. SUVR_50-70_ measures yielded comparable estimates to DVR, with ICC=0.89 (SD 0.03), range 0.82 in medial frontal cortex to 0.93 in putamen (p’s<0.001; **Supplementary Figure 1b, Supplementary Table 6**). Across brain regions, the mean difference was 6%±3%, which was comparable between menopausal groups (pre-menopause: 4%±3%, peri-menopause 6%±3%, post-menopause 7%±3%), and was deemed physiologically acceptable. This time window is also consistent with previous studies and observations that ^18^F-FES returns to sub-physiologic levels within ~1 hour post-injection^21,60^. Subsequent analyses are obtained using SUVR ROI data and voxel-wise SUVR_50-70_ images generated for all participants.

#### Multiparametric Analysis

Image processing was performed using a semi-automated pipeline^10-13^. ^18^F-FES PET, ASL and MRS images were realigned to the corresponding T1-weighted MRI using the surface-fitting Normalized Mutual Information algorithm implemented in SPM12^27^ running on Matlab 2021 (MathWorks; Natick.MA) to enable accurate sampling.

##### Regions-of-interest.

All ROIs except the manually delineated pituitary were quantified from MRI-coregistered ^18^F-FES images using the segmentation tools implemented in FreeSurfer 7.2 and Desikan-Killiany Atlas^25,26^ applied to the MRI. FreeSurfer was also used to derive total intracranial volume (TIV). Given its small size, the pituitary was only examined for ^18^F-FES effects. ^31^P-MRS data was processed via our proprietary XSOS written in IDL (Excelis Visual, Boulder, CO) using Hamming and Fermi k-space filters, a 7.5 mm center voxel shift, 20 Hz exponential filtering and zero-filling in time, x and y-domains prior to 3D Fast Fourier Transformation. A fixed first order phase of 4200° was applied to all spectra and data was automatically phased in zero order. The phospho-creatine (PCr) peak was set at 0.0 ppm and the central spectrum set as a reference, and susceptibility corrections performed throughout the data set. Baseline correction was applied to all other voxels in the chemical shift imaging (CSI) dataset by an experienced analyst (JPD). Peak area integration was performed around four well-resolved resonance peaks yielding PCr and total ATP (sum of α, β and γ-ATP moieties) expressed as percent area of the total phosphorous signal in the corresponding spectrum, and the PCr/ATP ratio (e.g. a marker of ATP re-synthesis) was computed. For^31^P-MRS analysis, medial temporal lobe regions were averaged, while hypothalamus and accumbens were excluded, due to size considerations.

##### Voxel-based analysis.

MRI scans were spatially normalized to the template-normalized tissue probabilistic map (TPM) image included in SPM12 conforming to the Montreal Neurological Institute (MNI) space, and processed using voxel-based morphometry (VBM) including image segmentation, Jacobian modulation, high-dimensional warping (DARTEL) of the segments, and application of an 8mm full-width at half maximum smoothing kernel^27^. Gray matter volume (GMV) segments were retained for statistical analysis. The MRI-coregistered ^18^F-FES SUVR and ASL scans were spatially normalized to the TPM image using MRI-derived subject-specific transformation matrices and smoothed at 10-mm FWHM^27^.

#### Covariates

Brain imaging analyses were adjusted by age and modality-specific confounders (cerebellar ^18^F-FES uptake; MRI total intracranial volume; global ASL blood flow; PCr/ATP). ^18^F-FES analyses were also adjusted by plasma E2 levels.

#### Statistical Analysis

Analyses were performed in SPSS v.25, R v.4.2.0 and SPM12. Clinical measures were examined with general linear models or chi-squared tests as appropriate. Our primary outcome was examination of ^18^F-FES SUVR (e.g. ER density) differences by menopause status. Additional outcomes included correlational analysis of ^18^F-FES SUVR with GMV, CBF, PCr/ATP and menopause symptom scores.

#### ER density by menopause status

##### ROI analysis.

Multivariate analysis of variance was used to test the statistical significance of the effect of menopause status (pre-, peri-, or post-menopausal) on ^18^F-FES SUVR in the set of target ROIs: pituitary, hypothalamus, accumbens, amygdala, hippocampus, cingulate, orbital and middle frontal gyrus. Post-hoc testing was performed to identify which pairwise comparisons in menopause status significantly differed across brain region measures. Regression models were constructed to obtain global P values for multivariate pair-wise outcomes across brain regions, followed by forest plot analysis for assessment of individual regions, at p<0.05. For completeness, SUVR in exploratory regions are provided in **Supplementary Table 7** for descriptive purposes.

##### Voxel-based analysis.

We used factorial models with post-hoc *t*-contrasts to test for ^18^F-FES differences between menopause groups, adjusting for age and cerebellar uptake. Statistical maps were constructed by applying a stringent voxel-level Gaussian random field theory–based threshold of p<0.05, cluster-level corrected for Family-Wise Type Error (FWE) within a binary masking image consisting of the full set of *a priori* defined regions used for sampling. Only clusters ≥16 voxels were considered significant. Anatomical location of significant clusters was described using Talairach coordinates after conversion from MNI space. Clusters of significant associations between ER density and menopause status were saved as binary masks (FES_mask_), and SUVR data were extracted from peak clusters using MarsBar 0.45 [https://marsbar-toolbox.github.io/download.html] for further analysis.

##### Prediction of menopause status:

(a) Standardized ROI and VBA-derived cluster SUVR were examined for menopause status separation using Cohen’s d effect size. After adjustment by age, the standardized pairwise mean differences between any two levels were expressed as Cohen’s d coefficients, where d≥0.8 reflects a large effect size. We used a conservative cut-off of 1.5 to identify the regions yielding the largest effect size in separating groups. (b) To gauge the degree to which ER density in these regions was predictive of menopause status, predictive models by means of multivariable logistic regressions were trained on a random 80% of the study sample, with 20% withheld as the testing set. Each model contained the binary outcome, pre- vs. post-menopause status. Our primary outcome was % accuracy in the testing set, defined as the proportion of correct predictions over total predictions. Global likelihood ratio tests were performed for each model at p<0.05.

#### Associations of ER density with brain biomarker measures

To characterize the relationship between ER density and additional imaging outcomes, correlation graphs are presented both for the overall study sample as well as by menopause status. The stratified analysis was performed to investigate hypothesized differences in the strength of the correlations throughout menopause. Stratification by menopause status also mitigates the effects of its confounding on the overall correlations.

##### ROI analysis.

We used multiple linear regressions to test for associations of regional ER density with GMV, CBF, and PCr/ATP adjusting by age and modality-specific confounders, at p<0.05.

##### Voxel-based analysis.

We used multiple linear regressions to test for voxel-wise associations of ER density with GMV and CBF in regions impacted by menopause status, and within peak clusters identified in analysis of menopause status, adjusting for age and modality-specific confounders. The latter analysis was done via the inclusive masking option at the t-map generation step, which allowed us to test for associations of ER density with GMV and CBF within the FES_mask_ image. For completeness, we used factorial analysis to test for voxel-wise associations of menopause status with GMV and CBF in the *a priori* masking image, thus independently of their associations with ER density. Results are reported in brain regions that survive the masking and the multiple comparisons adjustment.

#### Associations of ER density and menopause symptoms

We used a two-step approach to test for associations between ER density and menopause symptoms: (i) We computed voxel-wise linear regressionswith post-hoc t testsassessing associations between menopause symptom scores and ER density at p<0.05, cluster-level corrected within the masking image; (ii) we developed multivariable logistic regression models with menopause symptom occurrence as the binary outcome variable, and ER density as the exposure of interest, with age as a covariate. This analysis used both SUVR from peak clusters identified in (i) and target ROI. Analyses were performed across the entire cohort and within peri-menopausal and post-menopausal groups. Odds ratios (OR) were estimated, where a positive value denotes a positive association between ER density and presence of each menopause symptom. OR that exceeded 10 were capped at 10 to ensure visibility of positive associations < 10 in the figure.

## Figures and Tables

**Table 1:** ^18^F-FES estrogen receptor density in regions-of-interest by menopausal stage

	Pre-menopause	Peri-menopause	Post-menopause
Amygdala	1.011 (0.976, 1.045)	1.050 (1.025, 1.075)	1.055 (1.022, 1.089)
Anterior cingulate	1.164 (1.118, 1.211)	1.166 (1.132, 1.200)	1.222 (1.177, 1.267)
Caudate	1.109 (1.057, 1.161)	1.153 (1.116, 1.191)	1.167 (1.119, 1.219)
Hippocampus	1.212 (1.174, 1.249)	1.238 (1.21, 1.265)	1.251 (1.214, 1.288)
Hypothalamus	0.905 (0.864, 0.945)	0.972 (0.943, 1.001)	1.009 (0.969, 1.048)
Middle frontal gyrus	1.041 (1.007, 1.075)	1.075 (1.050, 1.100)	1.123 (1.088, 1.157)
Nucleus Accumbens	0.945 (0.903, 0.987)	0.999 (0.968, 1.029)	1.066 (1.025, 1.107)
Orbitofrontal gyrus	1.049 (1.010, 1.087)	1.088 (1.060, 1.116)	1.133 (1.095, 1.170)
Pituitary	3.729 (3.208, 4.25)	3.822 (3.444, 4.200)	4.045 (3.536, 4.554)
Putamen	1.282 (1.226, 1.339)	1.281 (1.24, 1.322)	1.297 (1.242, 1.352)
Thalamus	1.309 (1.262, 1.355)	1.309 (1.275, 1.343)	1.353 (1.308, 1.399)

Means and 95% confidence intervals by menopause status, adjusting for age. ^18^F-FES measures are standardized uptake value ratios (SUVR) to cerebellum.

**Table 2:** Voxel-wise menopause stage effects on brain ^18^F-FES estrogen receptor density

Cluster extent	Coordinates x,y,z	Z	P_FWE_*	P voxel	Anatomical Region	Brodmann Area
Post-menopause > Peri-menopause > Pre-menopause						
450	−31 11 36	4.39	0.007	< 0.001	Left Cerebrum, Frontal Lobe, Middle Frontal Gyrus	8
	−38 20 41	4.01		< 0.001	Left Cerebrum, Frontal Lobe, Middle Frontal Gyrus	8
259	17 44 25	4.37	0.021	< 0.001	Right Cerebrum, Frontal Lobe, Medial Frontal Gyrus	9
	15 35 32	3.20		< 0.001	Right Cerebrum, Frontal Lobe, Medial Frontal Gyrus	9
197	−50 −8 28	4.25	0.032	< 0.001	Left Cerebrum, Frontal Lobe, Precentral Gyrus	6
189	−29 −6 −8	3.87	0.034	< 0.001	Left Cerebrum, Sub-lobar, Lentiform Nucleus, Putamen	
1071	−6 42 – 21	3.83	< 0.001	< 0.001	Left Cerebrum, Frontal Lobe, Orbital Gyrus	11
	−6 16 – 11	3.79		< 0.001	Left Cerebrum, Limbic Lobe, Anterior Cingulate	25
	−6 20 – 17	3.67		< 0.001	Left Cerebrum, Frontal Lobe, Medial Frontal Gyrus	25
205	−38 27 25	3.70	0.030	< 0.001	Left Cerebrum, Frontal Lobe, Middle Frontal Gyrus	9
Post-menopause > Pre-menopause						
327	17 44 25	4.52	0.014	< 0.001	Right Cerebrum, Frontal Lobe, Medial Frontal Gyrus	9
	17 36 31	3.38		< 0.001	Right Cerebrum, Frontal Lobe, Medial Frontal Gyrus	9
522	−31 11 36	4.47	0.005	< 0.001	Left Cerebrum, Frontal Lobe, Middle Frontal Gyrus	8
	−38 20 41	4.09		< 0.001	Left Cerebrum, Frontal Lobe, Middle Frontal Gyrus	8
242	−50 −6 28	4.35	0.024	< 0.001	Left Cerebrum, Frontal Lobe, Precentral Gyrus	6
1126	−6 18 – 12	3.85	< 0.001	< 0.001	Left Cerebrum, Frontal Lobe, Anterior Cingulate gyrus	25
	−6 42 – 21	3.79		< 0.001	Left Cerebrum, Frontal Lobe, Orbital Gyrus	11
	−12 53 – 3	3.61		< 0.001	Left Cerebrum, Frontal Lobe, Superior Frontal Gyrus	10
242	−38 27 25	3.76	0.024	< 0.001	Left Cerebrum, Frontal Lobe, Middle Frontal Gyrus	9
Post-menopause > Peri-menopause						
228	46 33 15	3.91	0.005	< 0.001	Right Cerebrum, Frontal Lobe, Middle Frontal Gyrus	46
88	−34 12 41	3.65	0.014	< 0.001	Left Cerebrum, Frontal Lobe, Middle Frontal Gyrus	6
	−28 12 35	3.33		< 0.001	Left Cerebrum, Frontal Lobe, Middle Frontal Gyrus	8
51	−28 48 – 6	3.59	0.020	< 0.001	Left Cerebrum, Frontal Lobe, Middle Frontal Gyrus	10
45	17 44 25	3.57	0.022	< 0.001	Right Cerebrum, Frontal Lobe, Medial Frontal Gyrus	9
50	32 48 – 4	3.56	0.020	< 0.001	Right Cerebrum, Frontal Lobe, Middle Frontal Gyrus	10
36	−52 −6 29	3.56	0.024	< 0.001	Left Cerebrum, Frontal Lobe, Precentral Gyrus	6
29	−41 46 – 10	3.43	0.026	< 0.001	Left Cerebrum, Frontal Lobe, Middle Frontal Gyrus	11
228	46 33 15	3.91	0.005	< 0.001	Right Cerebrum, Frontal Lobe, Middle Frontal Gyrus	46
88	−34 12 41	3.65	0.014	< 0.001	Left Cerebrum, Frontal Lobe, Middle Frontal Gyrus	6
	−28 12 35	3.33		< 0.001	Left Cerebrum, Frontal Lobe, Middle Frontal Gyrus	8
Peri-menopause > Pre-menopause						
266	−28 −4 −8	4.10	0.011	< 0.001	Left Cerebrum, Sub-lobar, Lentiform Nucleus, Putamen	
	−32 −7 −12	3.63		< 0.001	Left Cerebrum, Limbic Lobe, Amygdala	
92	−49 –43 42	3.87	0.038	< 0.001	Left Cerebrum, Parietal Lobe, Inferior Parietal Lobule	40
82	−43 −15 49	3.80	0.042	< 0.001	Left Cerebrum, Frontal Lobe, Precentral Gyrus	4
74	12–62 21	3.69	0.045	< 0.001	Right Cerebrum, Occipital Lobe, Precuneus	31
65	−6 12–12	3.41	0.049	< 0.001	Left Cerebrum, Frontal Lobe, Anterior Cingulate Gyrus	25
110	−5 −41 32	3.37	0.033	< 0.001	Left Cerebrum, Limbic Lobe, Cingulate Gyrus	31

* P<0.05 cluster-level corrected for multiple comparisons. Results are adjusted by age, plasma estradiol levels, and cerebellar uptake.

## Data Availability

Methods, including de-identified Source Data files, will be made available in the online version of the paper.

## References

[1] McEwen B. Estrogen actions throughout the brain. Recent Prog Horm Res 57, 357–384, doi:10.1210/rp.57.1.357 (2002). 12017552

[2] McEwen B. S., Alves S. E., Bulloch K. & Weiland N. G. Ovarian steroids and the brain: implications for cognition and aging. Neurology 48, 8S–15S (1997). 10.1212/wnl.48.5_suppl_7.8s 9153161

[3] Brinton R. D., Yao J., Yin F., Mack W. J. & Cadenas E. Perimenopause as a neurological transition state. Nat Rev Endocrinol 11, 393–405, doi:10.1038/nrendo.2015.82 (2015). 26007613 PMC9934205

[4] Arevalo M. A., Azcoitia I. & Garcia-Segura L. M. The neuroprotective actions of oestradiol and oestrogen receptors. Nat Rev Neurosci 16, 17–29, doi:10.1038/nrn3856 (2015). 25423896

[5] Heldring N. Estrogen receptors: how do they signal and what are their targets. Physiol Rev 87, 905–931, doi:10.1152/physrev.00026.2006 (2007). 17615392

[6] McEwen B. S. Invited review: Estrogens effects on the brain: multiple sites and molecular mechanisms. J Appl Physiol (1985) 91, 2785–2801, doi:10.1152/jappl.2001.91.6.2785 (2001). 11717247

[7] Davis S. R. Menopause. Nat Rev Dis Primers 1, 15004, doi:10.1038/nrdp.2015.4 (2015). 27188659

[8] Monteleone P., Mascagni G., Giannini A., Genazzani A. R. & Simoncini T. Symptoms of menopause – global prevalence, physiology and implications. Nature Reviews Endocrinology 14, 199–215, doi:10.1038/nrendo.2017.180 (2018). 29393299

[9] Morrison J. H., Brinton R. D., Schmidt P. J. & Gore A. C. Estrogen, menopause, and the aging brain: how basic neuroscience can inform hormone therapy in women. J Neurosci 26, 10332–10348, doi:10.1523/JNEUROSCI.3369-06.2006 (2006). 17035515 PMC6674699

[10] Mosconi L. Sex differences in Alzheimer risk Brain imaging of endocrine vs chronologic aging. Neurology 89, 1382–1390 (2017). 28855400 10.1212/WNL.0000000000004425 PMC5652968

[11] Mosconi L., Rahman A., Diaz I., Wu X., Scheyer O., Hristov H., Vallabhajosula S., Isaacson R., Leon M., Brinton R. Increased Alzheimer's risk during the menopause transition: A 3-year longitudinal study. PloS one, 13(12): e0207885 (2018). 30540774 10.1371/journal.pone.0207885 PMC6291073

[12] Mosconi L. Menopause impacts human brain structure, connectivity, energy metabolism, and amyloid-beta deposition. Sci Rep 11, 10867, doi:10.1038/s41598-021-90084-y (2021). 34108509 PMC8190071

[13] Rahman A. Sex-driven modifiers of Alzheimer risk. Neurology 95, e166, doi:10.1212/WNL.0000000000009781 (2020). 32580974 PMC7455325

[14] Comasco E., Frokjaer V. G. & Sundström-Poromaa I. Functional and molecular neuroimaging of menopause and hormone replacement therapy. Front Neurosci 8, 388, doi:10.3389/fnins.2014.00388 (2014). 25538545 PMC4259109

[15] Maki P. M. The timing of estrogen therapy after ovariectomy–implications for neurocognitive function. Nature Clinical Practice Endocrinology & Metabolism 4, 494+ (2008). 10.1038/ncpendmet0901 18648334

[16] Mintun M. Breast cancer: PET imaging of estrogen receptors. 169, 45–48 (1988). 10.1148/radiology.169.1.3262228 3262228

[17] Katzenellenbogen J. A. The quest for improving the management of breast cancer by functional imaging: The discovery and development of 16alpha-[(18)F]fluoroestradiol (FES), a PET radiotracer for the estrogen receptor, a historical review. Nucl Med Biol 92, 24–37, doi:10.1016/j.nucmedbio.2020.02.007 (2021). 32229068 PMC7442693

[18] Liao G. J., Clark A. S., Schubert E. K. & Mankoff D. A. 18F-Fluoroestradiol PET: Current Status and Potential Future Clinical Applications. Journal of Nuclear Medicine 57, 1269–1275 (2016). 27307345 10.2967/jnumed.116.175596

[19] Moresco R. M. Systemic and cerebral kinetics of 16 alpha [18F]fluoro-17 beta-estradiol: a ligand for the in vivo assessment of estrogen receptor binding parameters. J Cereb Blood Flow Metab 15, 301–311, doi:10.1038/jcbfm.1995.35 (1995). 7860663

[20] Khayum M. A., de Vries E. F., Glaudemans A. W., Dierckx R. A. & Doorduin J. In vivo imaging of brain estrogen receptors in rats: a 16alpha-18F-fluoro-17beta-estradiol PET study. J Nucl Med 55, 481–487, doi:10.2967/jnumed.113.128751 (2014). 24481026

[21] Paquette M. Cross-Species Physiological Assessment of Brain Estrogen Receptor Expression Using (18)F-FES and (18)F-4FMFES PET Imaging. Mol Imaging Biol 22, 1403–1413, doi:10.1007/s11307-020-01520-w (2020). 32699974 PMC8080271

[22] Pareto D., Alvarado M., Hanrahan S. M. & Biegon A. In vivo occupancy of female rat brain estrogen receptors by 17beta-estradiol and tamoxifen. Neuroimage 23, 1161–1167, doi:10.1016/j.neuroimage.2004.07.036 (2004). 15528115

[23] Iqbal R., Menke-van der Houven van Oordt C. W., Oprea-Lager D. E. & Booij J. [(18)F]FES uptake in the pituitary gland and white matter of the brain. Eur J Nucl Med Mol Imaging 48, 3009–3010, doi:10.1007/s00259-021-05281-8 (2021). 33730173 PMC8263391

[24] Hattersley G., David F., Harris A.W., Clarkin, Banks, M.K., Williams G., Glaudemans A., Doorduin J., Koole M., de Vries E., Lyttle R. RAD1901, a novel tissue-selective estrogen receptor degrader demonstrates estrogen receptor engagement in a phase 1 clinical study. Cancer Res, OT2-1-20 (2015).

[25] Desikan R. S. An automated labeling system for subdividing the human cerebral cortex on MRI scans into gyral based regions of interest. NeuroImage 31, 968–980, doi:10.1016/j.neuroimage.2006.01.021 (2006). 16530430

[26] Fischl B. FreeSurfer. NeuroImage 62, 774–781, doi:10.1016/j.neuroimage.2012.01.021 (2012). 22248573 PMC3685476

[27] Ashburner J. & Friston K. J. Voxel-based morphometry--the methods. Neuroimage 11, 805–821, doi:10.1006/nimg.2000.0582 (2000). 10860804

[28] Osterlund M. K. & Hurd Y. L. Estrogen receptors in the human forebrain and the relation to neuropsychiatric disorders. Prog Neurobiol 64, 251–267, doi:10.1016/s0301-0082(00)00059-9 (2001). 11240308

[29] Gonzalez M. Distribution patterns of estrogen receptor alpha and beta in the human cortex and hippocampus during development and adulthood. J Comp Neurol 503, 790–802, doi:10.1002/cne.21419 (2007). 17570500

[30] Mitra S. W. Immunolocalization of estrogen receptor beta in the mouse brain: comparison with estrogen receptor alpha. Endocrinology 144, 2055–2067, doi:10.1210/en.2002-221069 (2003). 12697714

[31] Barth C., Villringer A. & Sacher J. Sex hormones affect neurotransmitters and shape the adult female brain during hormonal transition periods. Front Neurosci 9, 37, doi:10.3389/fnins.2015.00037 (2015). 25750611 PMC4335177

[32] Maioli S., Leander K., Nilsson P. & Nalvarte I. Estrogen receptors and the aging brain. Essays Biochem 65, 913–925, doi:10.1042/EBC20200162 (2021). PMC8628183 34623401

[33] Peterson L. M. Factors influencing the uptake of 18F-fluoroestradiol in patients with estrogen receptor positive breast cancer. Nucl Med Biol 38, 969–978, doi:10.1016/j.nucmedbio.2011.03.002 (2011). 21982568 PMC4108284

[34] Wilson M. E., Westberry J. M. & Prewitt A. K. Dynamic regulation of estrogen receptor-alpha gene expression in the brain: a role for promoter methylation? Front Neuroendocrinol 29, 375–385, doi:10.1016/j.yfrne.2008.03.002 (2008). 18439661 PMC2460564

[35] Caruso D. Effect of short-and long-term gonadectomy on neuroactive steroid levels in the central and peripheral nervous system of male and female rats. J Neuroendocrinol 22, 1137–1147, doi:10.1111/j.1365-2826.2010.02064.x (2010). 20819120

[36] Nilsen J. & Brinton R. D. Mitochondria as therapeutic targets of estrogen action in the central nervous system. Curr Drug Targets CNS Neurol Disord 3, 297–313, doi:10.2174/1568007043337193 (2004). 15379606

[37] Yin F. The perimenopausal aging transition in the female rat brain: decline in bioenergetic systems and synaptic plasticity. Neurobiol Aging 36, 2282–2295, doi:10.1016/j.neurobiolaging.2015.03.013 (2015). 25921624 PMC4416218

[38] Wang Y. Midlife Chronological and Endocrinological Transitions in Brain Metabolism: System Biology Basis for Increased Alzheimer’s Risk in Female Brain. Sci Rep 10, 8528, doi:10.1038/s41598-020-65402-5 (2020). 32444841 PMC7244485

[39] Ferretti M. T. Sex differences in Alzheimer disease - the gateway to precision medicine. Nature reviews. Neurology 14, 457–469, doi:10.1038/s41582-018-0032-9 (2018). 29985474

[40] Brass L. M. Hormone replacement therapy and stroke: clinical trials review. Stroke 35, 2644–2647, doi:10.1161/01.STR.0000143218.20061.ac (2004). 15459443

[41] Jett S. Ovarian steroid hormones: A long overlooked but critical contributor to brain aging and Alzheimer's disease. Front Aging Neurosci 14, 948219, doi:10.3389/fnagi.2022.948219 (2022). 35928995 PMC9344010

[42] Lobo R. A. Hormone-replacement therapy: current thinking. Nature Reviews Endocrinology 13, 220–231, doi:10.1038/nrendo.2016.164 (2017). 27716751

[43] Brinton R. D. The healthy cell bias of estrogen action: mitochondrial bioenergetics and neurological implications. Trends Neurosci 31, 529–537, doi:10.1016/j.tins.2008.07.003 (2008). 18774188 PMC10124615

[44] Henderson V. W. & Rocca W. A. (AAN Enterprises, 2012).

[45] Scott E., Zhang Q. G., Wang R., Vadlamudi R. & Brann D. Estrogen neuroprotection and the critical period hypothesis. Front Neuroendocrinol 33, 85–104, doi:10.1016/j.yfrne.2011.10.001 (2012). 22079780 PMC3288697

[46] Ito H., Hietala J., Blomqvist G., Halldin C. & Farde L. Comparison of the transient equilibrium and continuous infusion method for quantitative PET analysis of [11C]raclopride binding. J Cereb Blood Flow Metab 18, 941–950, doi:10.1097/00004647-199809000-00003 (1998). 9740097

[47] Logan J. Distribution volume ratios without blood sampling from graphical analysis of PET data. J Cereb Blood Flow Metab 16, 834–840, doi:10.1097/00004647-199609000-00008 (1996). 8784228

[48] Seo S. Noninvasive bi-graphical analysis for the quantification of slowly reversible radioligand binding. Phys Med Biol 61, 6770–6790, doi:10.1088/0031-9155/61/18/6770 (2016). 27580316

[49] Schubert J., Tonietto M., Turkheimer F., Zanotti-Fregonara P. & Veronese M. Supervised clustering for TSPO PET imaging. Eur J Nucl Med Mol Imaging 49, 257–268, doi:10.1007/s00259-021-05309-z (2021). 33779770 PMC8712290

[50] Mosconi L. Sex differences in Alzheimer risk: Brain imaging of endocrine vs chronologic aging. Neurology 89, 1382–1390, doi:10.1212/wnl.0000000000004425 (2017). 28855400 PMC5652968

[51] Mosconi L. Increased Alzheimer's risk during the menopause transition: A 3-year longitudinal brain imaging study. PLoS One 13, e0207885, doi:10.1371/journal.pone.0207885 (2018). 30540774 PMC6291073

[52] Harlow S. D. Executive summary of the Stages of Reproductive Aging Workshop + 10: addressing the unfinished agenda of staging reproductive aging. Menopause 19, 387–395, doi:10.1097/gme.0b013e31824d8f40 (2012). 22343510 PMC3340903

[53] Becker J. B. Strategies and Methods for Research on Sex Differences in Brain and Behavior. Endocrinology 146, 1650–1673, doi:10.1210/en.2004-1142 (2005). 15618360

[54] Wang J. Arterial transit time imaging with flow encoding arterial spin tagging (FEAST). Magn Reson Med 50, 599–607, doi:10.1002/mrm.10559 (2003). 12939768

[55] Knott K. E. Simplified and automatic one-pot synthesis of 16α-[18F]fluoroestradiol without high-performance liquid chromatography purification. Journal of Labelled Compounds and Radiopharmaceuticals 54, 749–753, doi:10.1002/jlcr.1916 (2011).

[56] Oh S. J. The automatic production of 16alpha-[18F]fluoroestradiol using a conventional [18F]FDG module with a disposable cassette system. Appl Radiat Isot 65, 676–681, doi:10.1016/j.apradiso.2006.06.016 (2007). 16963265

[57] Strother S. C. The quantitative evaluation of functional neuroimaging experiments: the NPAIRS data analysis framework. Neuroimage 15, 747–771, doi:10.1006/nimg.2001.1034 (2002). 11906218

[58] Tzourio-Mazoyer N. Automated anatomical labeling of activations in SPM using a macroscopic anatomical parcellation of the MNI MRI single-subject brain. Neuroimage 15, 273–289, doi:10.1006/nimg.2001.0978 (2002). 11771995

[59] MacMaster F. P. Pituitary volume in pediatric obsessive-compulsive disorder. Biol Psychiatry 59, 252–257, doi:10.1016/j.biopsych.2005.06.028 (2006). 16140279

[60] Paquette M. Improved Estrogen Receptor Assessment by PET Using the Novel Radiotracer (18)F-4FMFES in Estrogen Receptor-Positive Breast Cancer Patients: An Ongoing Phase II Clinical Trial. J Nucl Med 59, 197–203, doi:10.2967/jnumed.117.194654 (2018). 28798032 PMC6910621

